# Association of methylenetetrahydrofolate reductase C677T polymorphism and serum lipid levels in the Guangxi Bai Ku Yao and Han populations

**DOI:** 10.1186/1476-511X-9-123

**Published:** 2010-10-27

**Authors:** Lin Zhang, Rui-Xing Yin, Wan-Ying Liu, Lin Miao, Dong-Feng Wu, Lynn Htet Htet Aung, Xi-Jiang Hu, Xiao-Li Cao, Jin-Zhen Wu, Shang-Ling Pan

**Affiliations:** 1 Department of Cardiology, Institute of Cardiovascular Diseases, the First Affiliated Hospital, Guangxi Medical University, 22 Shuangyong Road, Nanning 530021, Guangxi, China; 2 Department of Pathophysiology, School of Premedical Sciences, Guangxi Medical University, Nanning 530021, Guangxi, China

## Abstract

**Background:**

The association of methylenetetrahydrofolate reductase (MTHFR) gene polymorphism and serum lipid profiles is still controversial in diverse ethnics. Bai Ku Yao is an isolated subgroup of the Yao minority in China. The aim of the present study was to eveluate the association of MTHFR C677T polymorphism and several environmental factors with serum lipid levels in the Guangxi Bai Ku Yao and Han populations.

**Methods:**

A total of 780 subjects of Bai Ku Yao and 686 participants of Han Chinese were randomly selected from our previous stratified randomized cluster samples. Genotyping of the MTHFR C677T was performed by polymerase chain reaction and restriction fragment length polymorphism combined with gel electrophoresis, and then confirmed by direct sequencing.

**Results:**

The levels of serum total cholesterol (TC), high-density lipoprotein cholesterol (HDL-C), low-density lipoprotein cholesterol (LDL-C), apolipoprotein (Apo) AI and ApoB were lower in Bai Ku Yao than in Han (*P *< 0.05-0.001). The frequency of C and T alleles was 77.4% and 22.6% in Bai Ku Yao, and 60.9% and 39.1% in Han (*P *< 0.001); respectively. The frequency of CC, CT and TT genotypes was 58.7%, 37.3% and 4.0% in Bai Ku Yao, and 32.6%, 56.4% and 11.0% in Han (*P *< 0.001); respectively. The levels of TC and LDL-C in both ethnic groups were significant differences among the three genotypes (*P *< 0.05-0.01). The T allele carriers had higher serum TC and LDL-C levels than the T allele noncarriers. The levels of ApoB in Han were significant differences among the three genotypes (*P *< 0.05). The T allele carriers had higher serum ApoB levels as compared with the T allele noncarriers. The levels of TC, TG and LDL-C in Bai Ku Yao were correlated with genotypes (*P *< 0.05-0.001), whereas the levels of LDL-C in Han were associated with genotypes (*P *< 0.001). Serum lipid parameters were also correlated with sex, age, body mass index, alcohol consumption, cigarette smoking, and blood pressure in the both ethnic groups.

**Conclusions:**

The differences in serum TC, TG, LDL-C and ApoB levels between the two ethnic groups might partly result from different genotypic and allelic frequencies of the MTHFR C677T or different MTHFR gene-enviromental interactions.

## Introduction

Disorders of lipid metabolism such as elevated serum levels of total cholesterol (TC) [1], triglyceride (TG) [2], low-density lipoprotein cholesterol (LDL-C) [3], and apolipoprotein (Apo) B [4], or low levels of high-density lipoprotein cholesterol (HDL-C) and ApoAI [4-6] have been considered to be important risk factors in the pathogenesis of atherosclerosis and coronary artery disease (CAD). It is well recognized that dyslipidemia is a complex trait caused by multiple environmental and genetic factors and their interactions [7-12]. Family history and twin studies have shown that genetic polymorphism could account for 40-60% of the interindividual variation in plasma lipid phenotypes [13-15]. The 5-10-methylenetetrahydrofolate reductase (MTHFR) gene that plays a role in vascular homeostasis has been considered to be good candidate for susceptibility to dyslipidemia.

The C677T polymorphism in the MTHFR gene, which encodes an enzyme involved in remethylation of homocysteine to methionine, leads to increased thermolability and impaired enzymatic activity. In its homozygous form, this variant possesses a reduced overall enzyme activity to less than 30% of normal, resulting on increased serum homocysteine levels [16,17]. The MTHFR 677T allele has been found to be associated with modifications of serum lipid concentrations [18-26] and the risk of CAD [26-30] in some studies but not in others [31-36].

Han is the largest ethnic group and Yao is the eleventh largest minority among the 55 minority groups in China according to the population size. Bai Ku Yao (White-trouser Yao), an isolated subgroup of the Yao minority, is named so because all the men wear white knee-length knickerbockers. The population size is about 30000. Because of isolation from the other ethnic groups, the special customs and cultures including their clothing, intra-ethnic marriages, dietary habits, and corn wine and rum intakes are still completely preserved to the present day. In previous epidemiological studies, we found that serum lipid levels were lower in Bai Ku Yao than in Han Chinese from the same village [7,8]. This ethnic difference in serum lipid profiles is still not well known. We hypothesized that there may be significant differences in some genetic factors between the two ethnic groups. Therefore, the aim of the present study was to detect the association of MTHFR C677T polymorphism and several environmental factors with serum lipid levels in the Guangxi Bai Ku Yao and Han populations.

## Materials and methods

### Study population

A total of 780 subjects of Bai Ku Yao who reside in Lihu and Baxu villages in Nandan County, Guangxi Zhuang Autonomous Region, People's Republic of China were randomly selected from our previous stratified randomized cluster samples [7,8]. The ages of the subjects ranged from 15 to 80 years, with an average age of 40.24 ± 13.07 years. There were 381 males (48.85%) and 399 females (51.15%). All subjects were rural agricultural workers. The subjects accounted for 2.60% of total Bai Ku Yao population. During the same period, a total of 686 people of Han Chinese who reside in the same villages were also randomly selected from our previous stratified randomized cluster samples [7,8]. The average age of the subjects was 39.61 ± 14.67 years (range 15 to 80). There were 342 men (49.85%) and 344 women (50.15%). All of them were also rural agricultural workers. All study subjects were essentially healthy and had no evidence of any chronic illness, including hepatic, renal, or thyroid. The participants with a history of heart attack or myocardial infarction, stroke, congestive heart failure, diabetes or fasting blood glucose ≥ 7.0 mmol/L determined by glucose meter have been excluded. The participants were not taking medications known to affect serum lipid levels (lipid-lowering drugs such as statins or fibrates, beta-blockers, diuretics, or hormones). The present study was approved by the Ethics Committee of the First Affiliated Hospital, Guangxi Medical University. Informed consent was obtained from all subjects after they received a full explanation of the study.

### Epidemiological survey

The survey was carried out using internationally standardized methods, following a common protocol [37]. Information on demographics, socioeconomic status, and lifestyle factors was collected with standardized questionnaires. The alcohol information included questions about the number of liangs (about 50 g) of rice wine, corn wine, rum, beer, or liquor consumed during the preceding 12 months. Alcohol consumption was categorized into groups of grams of alcohol per day: < 25 and ≥ 25. Smoking status was categorized into groups of cigarettes per day: < 20 and ≥ 20. At the physical examination, several anthropometric parameters, such as height, weight, and waist circumference were measured. Sitting blood pressure was measured three times with the use of a mercury sphygmomanometer after the subjects had a 5-minute rest, and the average of the three measurements was used for the level of blood pressure. Systolic blood pressure was determined by the first Korotkoff sound, and diastolic blood pressure by the fifth Korotkoff sound. Body weight, to the nearest 50 grams, was measured using a portable balance scale. Subjects were weighed without shoes and in a minimum of clothing. Height was measured, to the nearest 0.5 cm, using a portable steel measuring device. From these two measurements body mass index (BMI, kg/m^2^) was calculated. Waist circumference was measured with a nonstretchable measuring tape, at the level of the smallest area of the waist, to the nearest 0.1 cm.

### Biochemical analysis

A venous blood sample of 8 ml was obtained from all subjects between 8 and 11 AM, after at least 12 hours of fasting, from a forearm vein after venous occlusion for few seconds in a sitting position. A part of the sample (3 ml) was collected into glass tubes and allowed to clot at room temperature, and used to determine serum lipid levels. Another part of the sample (5 ml) was transferred to tubes with anticoagulate solution (4.80 g/L citric acid, 14.70 g/L glucose, and 13.20 g/L tri-sodium citrate) and used to extract DNA. Immediately following clotting serum was separated by centrifugation for 15 minutes at 3000 rpm. The levels of TC, TG, HDL-C, and LDL-C in samples were determined by enzymatic methods with commercially available kits, Tcho-1, TG-LH (RANDOX Laboratories Ltd., Ardmore, Diamond Road, Crumlin Co. Antrim, United Kingdom, BT29 4QY), Cholestest N HDL, and Cholestest LDL (Daiichi Pure Chemicals Co., Ltd., Tokyo, Japan); respectively. Serum ApoAI and ApoB levels were detected by the immunoturbidimetric immunoassay using a commercial kit (RANDOX Laboratories Ltd.). All determinations were performed with an autoanalyzer (Type 7170A; Hitachi Ltd., Tokyo, Japan) in the Clinical Science Experiment Center of the First Affiliated Hospital, Guangxi Medical University [7,8].

### DNA amplification and genotyping

Genomic DNA was isolated from peripheral blood leukocytes using the phenol-chloroform method [11,12]. The extracted DNA was stored at 4°C until analysis. Genotyping of the MTHFR C677T was performed by polymerase chain reaction and restriction fragment length polymorphism (PCR-RFLP) [38-40]. PCR amplification was performed using 5'-CAAAGGCCACCCCGAAGC-3' and 5'-AGGACGGTGCGGTGAGAGTG-3' (Sangon, Shanghai, People's Republic of China) as the forward and reverse primer pairs; respectively. Each amplification reaction was performed in a total volume of 25 μL, containing 10 × PCR buffer (1.8 mM MgCl_2_) 2.5 μL, 1 U *Taq *polymerase, 2.5 mmol/L of each dNTP (Tiangen, Beijing, People's Republic of China) 2.0 μL, 5 pmol/L of each primer (0.5 μL) and 2 μL of genomic DNA, processing started with 94°C for 5 min and 34 cycles at 94°C for 45 s, 61.5°C for 40 s and 72°C for 50 s. This was followed by a final extension at 72°C for 7 min. Then 5 U of *Hinf*I enzyme was added directly to the PCR products (5 μL) and digested at 37°C overnight. After restriction enzyme digestion of the amplified DNA, genotypes were identified by electrophoresis on 2% agarose gels and visualized with ethidium-bromide staining ultraviolet illumination. Genotypes were scored by an experienced reader blinded to epidemiological data and serum lipid levels. Six samples (CC, CT and TT genotypes in two; respectively) detected by the PCR-RFLP were also confirmed by direct sequencing. The PCR products were purified by low melting point gel electrophoresis and phenol extraction, and then the DNA sequences were analyzed in Shanghai Sangon Biological Engineering Technology & Services Co., Ltd., People's Republic of China.

### Diagnostic criteria

The normal values of serum TC, TG, HDL-C, LDL-C, ApoAI, ApoB levels, and the ratio of ApoAI to ApoB in our Clinical Science Experiment Center were 3.10-5.17, 0.56-1.70, 0.91-1.81, 2.70-3.20 mmol/L, 1.00-1.78, 0.63-1.14 g/L, and 1.00-2.50; respectively. The individuals with TC > 5.17 mmol/L and/or TG > 1.70 mmol/L were defined as hyperlipidemic [7,8]. Hypertension was diagnosed according to the criteria of 1999 World Health Organization-International Society of Hypertension Guidelines for the management of hypertension [41,42]. The diagnostic criteria of overweight and obesity were according to the Cooperative Meta-analysis Group of China Obesity Task Force. Normal weight, overweight and obesity were defined as a BMI < 24, 24-28, and > 28 kg/m^2^; respectively [43].

### Statistical analyses

Epidemiological data were recorded on a pre-designed form and managed with Excel software. All statistical analyses were done with the statistical software package SPSS 13.0 (SPSS Inc., Chicago, Illinois). Quantitative variables were expressed as mean ± standard deviation (serum TG levels were presented as medians and interquartile ranges). Qualitative variables were expressed as percentages. Allele frequency was determined via direct counting, and the standard goodness-of-fit test was used to test the Hardy-Weinberg equilibrium. Difference in genotype distribution between the groups was obtained using the chi-square test. The difference in general characteristics between Bai Ku Yao and Han was tested by the Student's unpaired *t*-test. The association of genotypes and serum lipid parameters was tested by analysis of covariance (ANCOVA). Sex, age, BMI, blood pressure, alcohol consumption, cigarette smoking were adjusted for the statistical analysis. In order to evaluate the association of serum lipid levels with genotypes and several environment factors, multivariate logistic regression analysis was also performed in the combined population of Bai Ku Yao and Han, Bai Ku Yao, and Han; respectively. A *P *value of less than 0.05 was considered statistically significant.

## Results

### General characteristics and serum lipid levels

Table 1 gives the general characteristics and serum lipid levels between the Bai Ku Yao and Han populations. The levels of BMI, systolic blood pressure, diastolic blood pressure, pulse pressure, serum TC, HDL-C, LDL-C, ApoAI and ApoB were lower in Bai Ku Yao than in Han Chinese (*P *< 0.05-0.001), whereas the percentage of subjects who smoked cigarettes was higher in Bai Ku Yao than in Han (*P *< 0.001). There were no significant differences in the levels of serum TG, the ratio of ApoAI to ApoB, age structure, the percentages of subjects who consumed alcohol, or the ratio of male to female between the two ethnic groups (*P *> 0.05 for all).

**Table 1:** The general characteristics and serum lipid levels between the Bai Ku Yao and Han populations

Parameter	Bai Ku Yao	Han Chinese	***t *(*χ***^**2**^**)**	*P*
Number	780	686	-	-
Male/female	381/399	342/344	0.148	0.714
Age (years)	40.24 ± 13.07	39.61 ± 14.67	0.866	0.386
Body mass index (kg/m^2^)	22.05 ± 2.31	22.51 ± 2.74	-3.467	0.001
Systolic blood pressure (mmHg)	116.55 ± 15.39	120.63 ± 15.94	-4.985	0.000
Diastolic blood pressure (mmHg)	74.78 ± 9.30	76.29 ± 10.04	-2.983	0.003
Pulse pressure (mmHg)	41.77 ± 11.44	44.40 ± 10.94	-4.478	0.000
Cigarette smoking [n(%)]				
Nonsmoker	531(68.1)	514(74.9)		
< 20 cigarettes/day	128(16.4)	52(7.6)		
≥20 cigarettes/day	121(15.5)	120(17.5)	26.451	0.000
Alcohol consumption [n(%)]				
Nondrinker	443(56.8)	401(58.4)		
< 25 g/day	240(30.8)	179(26.1)		
≥25 g/day	107(13.4)	106(15.5)	3.666	0.160
Total cholesterol (mmol/L)	4.26 ± 0.93	4.72 ± 0.93	-9.372	0.000
Triglyceride (mmol/L)	0.97(0.71)	1.01(0.77)	-1.670	0.095
HDL-C (mmol/L)	1.65 ± 0.75	1.92 ± 0.50	-11.272	0.000
LDL-C (mmol/L)	2.50 ± 0.75	2.59 ± 0.72	-2.350	0.019
Apolipoprotein (Apo) AI (g/L)	1.29 ± 0.32	1.42 ± 0.23	-8.732	0.000
ApoB (g/L)	0.83 ± 0.23	0.90 ± 0.21	-6.656	0.000
ApoAI/ApoB	1.69 ± 0.78	1.64 ± 0.43	1.703	0.089

HDL-C, high-density lipoprotein cholesterol; LDL-C, low-density lipoprotein cholesterol. The value of TG was presented as median (interquartile range). The difference between the two ethnic groups was determined by the Wilcoxon-Mann-Whitney test.

### Results of electrophoresis and genotyping

**Figure 1:**
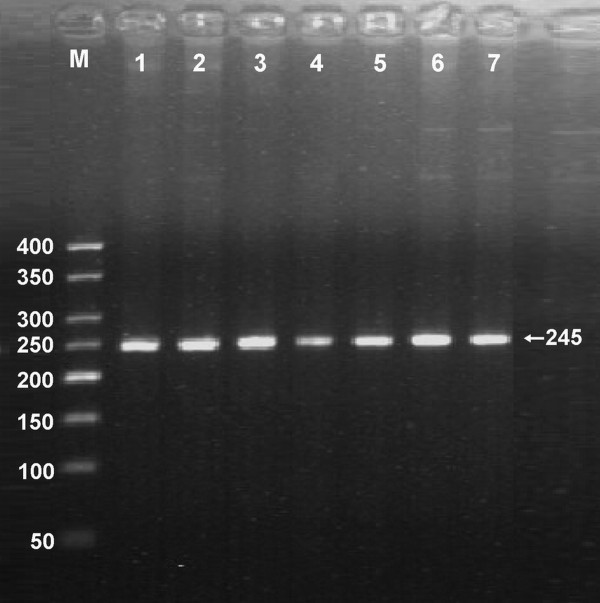
**Electrophoresis of PCR products of the samples**. Lane M, 50 bp marker ladder; lanes 1-7, samples. The 245 bp bands are the target genes.

**Figure 2:**
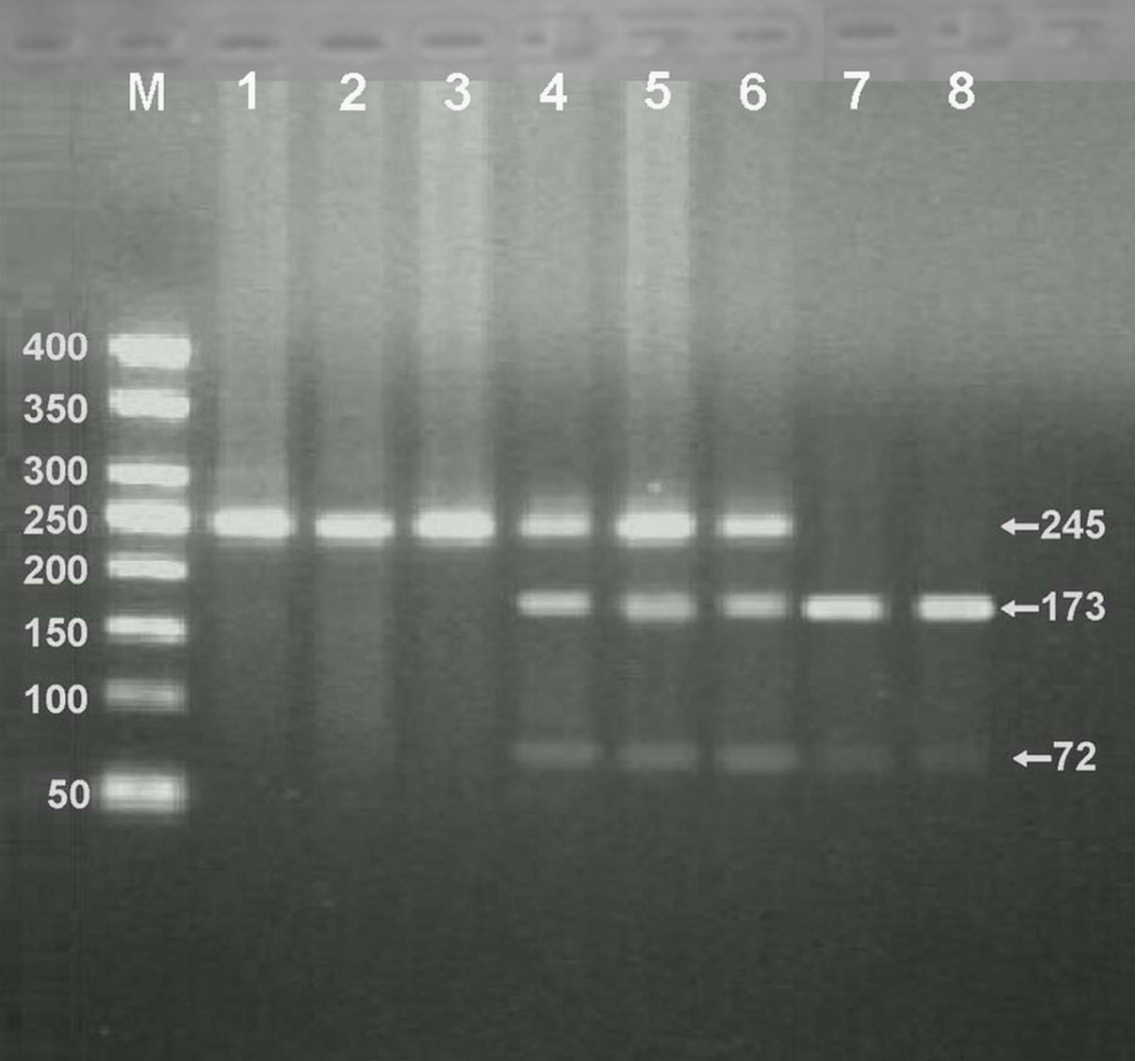
**Genotyping of the MTHFR C677T polymorphism**. Lane M, 50 bp marker ladder; lanes 1 and 2, CC genotype (245 bp); lane 3 and 4, CT genotype (245-, 173- and 72-bp); and lanes 5 and 6, TT genotype (173- and 72-bp).

After the genomic DNA of the samples was amplified by PCR and imaged by 2% agarose gel electrophoresis, the purpose gene of 254 bp nucleotide sequences could be found in all samples (Figure 1). The genotypes identified were named according to the presence or absence of the enzyme restriction sites, when a C to T transversion at nucleotide position 677 of the MTHFR gene. The presence of the cutting site indicates the T allele, while its absence indicates the C allele. Thus, the CC genotype is homozygote for the absence of the site (band at 254 bp), CT genotype is heterozygote for the absence and presence of the site (bands at 254-, 173- and 72-bp), and TT genotype is homozygote for the presence of the site (bands at 173- and 72- bp; Figure 2).

### Genotypic and allelic frequencies

The frequency of MTHFR C677T alleles and genotypes is shown in Table 2. The frequency of C and T alleles was 77.4% and 22.6% in Bai Ku Yao, and 60.9% and 39.1% in Han (*P *< 0.001); respectively. The frequency of CC, CT and TT genotypes was 58.7%, 37.3% and 4.0% in Bai Ku Yao, and 32.6%, 56.4% and 11.0% in Han (*P *< 0.001); respectively. There was no significant difference in the genotypic and allelic frequencies between males and females in both ethnic groups.

**Table 2:** Genotypic and allelic frequencies of the MTHFR C677T between the Bai Ku Yao and Han populations [n(%)]

Group	n	Genotype			Allele	
			
		CC	CT	TT	C	T
Bai Ku Yao	780	458(58.7)	291(37.3)	31(4.0)	1207(77.4)	353(22.6)
Han Chinese	686	224(32.6)	387(56.4)	75(11.0)	835(60.9)	537(39.1)
*χ*^2^	-	106.6			94.142	
*P*	-	0.000			0.000	
Bai Ku Yao						
Male	381	228(59.8)	131(34.4)	22(5.8)	587(77.0)	175(23.0)
Female	399	230(57.6)	156(39.1)	13(3.3)	616(77.2)	182(22.8)
*χ*^2^	-	4.088			0.006	
*P*	-	0.130			0.952	
Han Chinese						
Male	342	107(31.3)	199(58.2)	36(10.5)	413(60.4)	271(39.6)
Female	344	117(34.0)	188(54.7)	39(11.3)	422(61.3)	266(38.7)
*χ*^2^	-	0.873			0.716	
*P*	-	0.928			0.740	

### Results of sequencing

**Figure 3:**
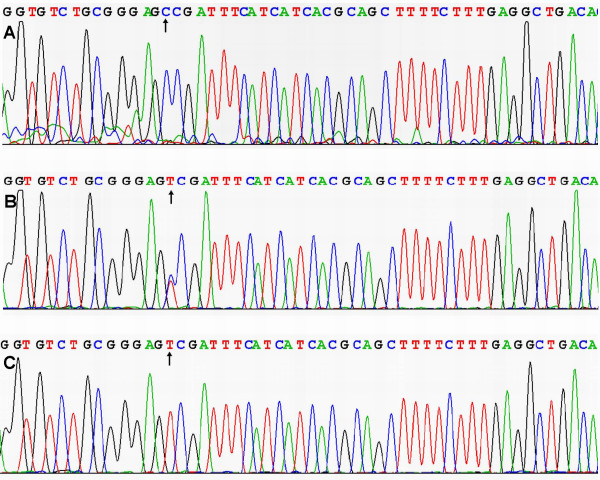
**A part of the nucleotide sequence of the MTHFR C677T locus**. (A) CC genotype; (B) CT genotype; (C) TT genotype.

The results were shown as CC, CT and TT genotypes by PCR-RFLP, the CC, CT and TT genotypes were also confirmed by sequencing (Figure 3); respectively.

### Genotypes and serum lipid levels

As shown in Table 3, the levels of TC and LDL-C were significant differences among the three genotypes in both ethnic groups (*P *< 0.05-0.01). The T allele carriers had higher serum TC and LDL-C levels than the T allele noncarriers. The levels of ApoB were also significant differences among the three genotypes in Han Chinese (*P *< 0.05). The T allele carriers had higher serum ApoB levels than the T allele noncarriers. There was no significant difference in the levels of TG, HDL-C, ApoAI and the ratio of ApoAI to ApoB among the three genotypes in both ethnic groups (*P *> 0.05 for all).

**Table 3:** Genotypic frequencies of the MTHFR C677T and serum lipid levels between the Bai Ku Yao and Han populations

Genotype	n	TC (mmol/L)	TG (mmol/L)	HDL-C (mmol/L)	LDL-C (mmol/L)	ApoAI (g/L)	ApoB (g/L)	ApoAI/ApoB
Bai Ku Yao								
CC	458	4.20 ± 0.85	0.97(0.73)	1.62 ± 0.40	2.45 ± 0.70	1.27 ± 3.15	0.82 ± 0.22	1.69 ± 0.81
CT	291	4.33 ± 1.06	0.94(0.68)	1.67 ± 0.44	2.57 ± 0.85^a^	1.30 ± 3.33	0.84 ± 0.25	1.70 ± 0.77
TT	31	4.52 ± 0.62	1.11(0.68)	1.75 ± 0.40	2.67 ± 0.44^a^	1.38 ± 0.34	0.86 ± 0.15	1.63 ± 0.40
*F*	-	2.807	0.932	1.993	3.299	1.884	0.592	0.091
*P*	-	0.061	0.334	0.137	0.037	0.153	0.554	0.913
CC	458	4.20 ± 0.85	0.97(0.73)	1.62 ± 0.40	2.45 ± 0.70	1.27 ± 0.31	0.82 ± 0.22	1.69 ± 0.81
CT/TT	322	4.34 ± 1.02	0.96(0.68)	1.68 ± 0.43	2.58 ± 0.81	1.31 ± 0.33	0.84 ± 0.24	1.69 ± 0.75
*F*	-	2.098	0.611	1.722	2.462	1.489	0.942	0.040
*P*	-	0.036	0.541	0.085	0.014	0.137	0.347	0.968
Male								
CC	228	4.20 ± 0.90	1.08(0.78)	1.64 ± 0.45	2.38 ± 0.75	1.32 ± 0.34	0.81 ± 0.22	1.82 ± 1.01
CT	131	4.33 ± 1.27	1.11(0.80)	1.67 ± 0.50	2.54 ± 1.01	1.34 ± 0.40	0.81 ± 0.26	1.87 ± 1.03
TT	22	4.63 ± 0.73	1.02(0.66)	1.80 ± 0.39	2.75 ± 0.54	1.37 ± 0.40	0.88 ± 0.18	1.61 ± 0.44
*F*	-	2.076	1.005	1.416	2.873	0.258	0.971	0.636
*P*	-	0.127	0.605	0.244	0.058	0.773	0.379	0.530
CC	228	4.20 ± 0.90	1.08(0.78)	1.64 ± 0.45	2.38 ± 0.75	1.32 ± 0.34	0.81 ± 0.22	1.82 ± 1.01
CT/TT	153	4.37 ± 1.21	1.07(0.80)	1.69 ± 0.48	2.58 ± 0.96	1.35 ± 0.39	0.82 ± 0.25	1.83 ± 0.97
*F*	-	1.619	0.105	1.108	2.139	0.701	0.536	0.064
*P*	-	0.106	0.916	0.269	0.033	0.484	0.592	0.949
Female								
CC	230	4.21 ± 0.80	0.92(0.69)	1.61 ± 0.35	2.51 ± 0.63	1.23 ± 0.25	0.83 ± 0.20	1.56 ± 0.51
CT	156	4.28 ± 0.83	0.82(0.65)	1.67 ± 0.39	2.57 ± 0.65	1.27 ± 0.25	0.85 ± 0.24	1.50 ± 0.38
TT	13	4.68 ± 0.66	1.65(0.95)^ac^	1.63 ± 0.34	2.87 ± 0.53	1.36 ± 0.29	0.91 ± 0.14	1.57 ± 0.42
*F*	-	2.363	9.541	1.024	2.097	2.433	0.841	0.108
*P*	-	0.095	0.008	0.360	0.124	0.089	0.432	0.897
CC	230	4.21 ± 0.80	0.92(0.69)	1.61 ± 0.35	2.51 ± 0.63	1.23 ± 0.25	0.83 ± 0.20	1.56 ± 0.51
CT/TT	156	4.32 ± 0.82	0.83(0.66)	1.66 ± 0.38	2.59 ± 0.64	1.28 ± 0.26	0.86 ± 0.23	1.56 ± 0.42
*F*	-	1.344	0.757	1.395	1.210	1.738	0.730	0.019
*P*	-	0.180	0.449	0.164	0.227	0.083	0.466	0.985
Han Chinese								
CC	224	4.56 ± 0.81	0.97(0.73)	1.91 ± 0.52	2.48 ± 0.66	1.40 ± 0.23	0.88 ± 0.20	1.67 ± 0.43
CT	387	4.77 ± 0.95^a^	1.02(0.79)	1.92 ± 0.48	2.64 ± 0.73^a^	1.42 ± 0.23	0.92 ± 0.21	1.61 ± 0.41
TT	75	4.88 ± 1.05^b^	1.02(0.78)	1.93 ± 0.53	2.69 ± 0.80^a^	1.42 ± 0.25	0.92 ± 0.23	1.65 ± 0.51
*F*	-	5.003	2.468	0.093	4.218	0.508	2.756	1.214
*P*	-	0.007	0.288	0.911	0.015	0.602	0.064	0.298
CC	224	4.56 ± 0.81	0.97(0.73)	1.90 ± 0.52	2.48 ± 0.66	1.40 ± 0.23	0.88 ± 0.20	1.67 ± 0.43
CT/TT	462	4.79 ± 0.97	1.02(0.79)	1.92 ± 0.48	2.65 ± 0.74	1.42 ± 0.24	0.92 ± 0.21	1.62 ± 0.43
*F*	-	3.036	1.538	0.347	2.853	1.007	2.346	1.399
*P*	-	0.002	0.124	0.708	0.004	0.314	0.019	0.162
Male								
CC	107	4.58 ± 0.86	1.03(0.73)	188 ± 0.57	2.45 ± 0.69	1.39 ± 0.26	0.88 ± 0.21	1.66 ± 0.46
CT	199	4.72 ± 0.94	1.02(0.80)	1.85 ± 0.49	2.61 ± 0.69	1.40 ± 0.24	0.91 ± 0.20	1.60 ± 0.41
TT	36	5.08 ± 1.05^bc^	1.06(0.80)	1.93 ± 0.54	2.79 ± 0.91^a^	1.43 ± 0.27	0.94 ± 0.24	1.64 ± 0.57
*F*	-	4.105	0.554	0.328	3.426	0.461	0.991	0.612
*P*	-	0.017	0.758	0.720	0.034	0.631	0.372	0.543
CC	107	4.58 ± 0.86	1.03(0.73)	188 ± 0.57	2.45 ± 0.69	1.39 ± 0.26	0.88 ± 0.21	1.66 ± 0.46
CT/TT	235	4.77 ± 0.96	1.02(0.80)	1.87 ± 0.50	2.64 ± 0.73	1.40 ± 0.25	0.91 ± 0.21	1.61 ± 0.44
*F*	-	1.807	0.710	0.168	2.160	0.188	1.128	0.966
*P*	-	0.072	0.478	0.867	0.032	0.851	0.260	0.335
Female								
CC	117	4.55 ± 0.76	0.92(0.74)	1.93 ± 0.46	2.50 ± 0.61	1.41 ± 0.19	0.87 ± 0.19	1.68 ± 0.41
CT	188	4.81 ± 1.02^a^	1.00(0.76)	1.98 ± 0.45	2.65 ± 0.78	1.44 ± 0.24	0.93 ± 0.22	1.63 ± 0.42
TT	39	4.80 ± 0.72	0.97(0.76)	1.97 ± 0.47	2.64 ± 0.62	1.44 ± 0.16	0.91 ± 0.19	1.65 ± 0.45
*F*	-	3.091	1.704	0.377	1.802	1.002	2.487	0.537
*P*	-	0.047	0.427	0.687	0.167	0.368	0.085	0.585
CC	117	4.55 ± 0.76	0.92(0.74)	1.93 ± 0.46	2.50 ± 0.61	1.41 ± 0.19	0.87 ± 0.19	1.68 ± 0.41
CT/TT	227	4.81 ± 0.97	1.00(0.76)	1.98 ± 0.45	2.65 ± 0.72	1.44 ± 0.23	0.92 ± 0.21	1.63 ± 0.42
*F*	-	2.489	1.288	0.864	1.892	1.400	2.191	0.979
*P*	-	0.013	0.198	0.388	0.059	0.162	0.029	0.328

TC, total cholesterol; TG, triglyceride; HDL-C, high-density lipoprotein cholesterol; LDL-C, low-density lipoprotein cholesterol; ApoAI, apolipoprotein AI; ApoB, apolipoprotein B; ApoAI/ApoB, the ratio of apolipoprotein AI to apolipoprotein B. The value of TG was presented as median (interquartile range). The difference in serum TG levels was determined by the Kruskal-Wallis test or the Wilcoxon-Mann-Whitney test. ^a^*P *< 0.05 and ^b^*P *< 0.05 in comparison with CC genotype and ^c^*P *< 0.05 in comparison with CT genotype in the same group

### Relative factors for serum lipid parameters

Multivariate logistic regression analysis showed that the levels of TC, TG and LDL-C were correlated with genotypes in Bai Ku Yao (*P *< 0.05-0.001), whereas the levels of LDL-C were correlated with genotypes in Han (*P *< 0.001). Serum lipid parameters were also correlated with sex, age, BMI, alcohol consumption, cigarette smoking, and blood pressure in both ethnic groups (Table 4).

**Table 4:** Correlative factors for serum lipid paramerers between the Bai Ku Yao and Han populations

Lipid	Relative factor	Regression coefficient	Standard error	***χ***^**2**^	*P*
Bai Ku Yao and Han					
TC	Sex	-0.732	0.156	21.958	0.000
	Age	0.011	0.005	5.847	0.016
	Alcohol consumption	-0.627	0.110	32.569	0.000
	Ethnic group	0.333	0.133	6.245	0.012
	Body mass index	0.154	0.026	35.384	0.000
TG	Cigarette smoking	0.322	0.108	8.865	0.003
	Genotype	0.169	0.026	23.201	0.000
HDL-C	Age	-0.035	0.014	6.392	0.011
	Cigarette smoking	0.601	0.245	6.037	0.014
	Ethnic group	-1.043	0.365	8.106	0.004
LDL-C	Age	0.013	0.006	3.875	0.049
	Cigarette smoking	-1.070	0.178	36.257	0.000
	Alcohol consumption	-1.243	0.181	47.134	0.000
	Genotype	0.164	0.034	23.437	0.000
ApoAI	Sex	0.745	0.149	25.013	0.000
	Alcohol consumption	-0.272	0.094	8.320	0.004
	Ethnic group	-0.498	0.084	21.805	0.000
ApoB	Alcohol consumption	-0.573	0.089	31.968	0.000
	Ethnic group	-0.435	0.116	14.142	0.000
	Sex	0.536	0.146	13.468	0.000
ApoAI/ApoB	Alcohol consumption	-0.250	0.089	7.973	0.005
	Ethnic group	0.593	0.103	32.808	0.000
	Genotype	-0.117	0.024	24.545	0.000
	Systolic blood pressure	-0.265	0.096	7.650	0.006
Bai Ku Yao					
TC	Sex	-4.320	0.567	57.972	0.000
	Alcohol consumption	-5.245	0.805	48.720	0.000
	Genotype	1.160	0.249	21.732	0.000
TG	Sex	0.693	0.259	7.155	0.007
	Body mass index	0.180	0.039	21.249	0.000
	Genotype	0.537	1.162	10.924	0.001
HDL-C	Age	-0.031	0.010	4.119	0.047
	Cigarette smoking	0.695	0.315	4.872	0.027
	Alcohol consumption	0.859	0.294	8.545	0.003
LDL-C	Age	0.040	0.015	6.971	0.008
	Genotype	0.357	0.156	5.244	0.022
ApoAI	Sex	1.021	0.217	22.085	0.000
	Alcohol consumption	-0.869	0.145	35.851	0.000
	Body mass index	-0.121	0.038	10.306	0.001
ApoB	Sex	0.781	0.253	9.557	0.002
	Alcohol consumption	-3.687	0.682	37.490	0.000
	Body mass index	-0.115	0.156	8.197	0.004
ApoAI/ApoB	Sex	1.049	0.209	25.098	0.000
	Body mass index	-0.105	0.035	9.141	0.002
Han Chinese					
TC	Age	0.027	0.007	16.531	0.000
	Body mass index	0.164	0.033	24.132	0.000
TG	Cigarette smoking	0.380	0.140	7.338	0.007
	Body mass index	0.232	0.037	38.897	0.000
HDL-C	Age	-0.060	0.031	3.865	0.049
LDL-C	Age	0.022	0.009	5.483	0.019
	Body mass index	0.215	0.043	24.399	0.000
	Genotype	0.503	0.133	14.276	0.000
ApoAI	Alcohol consumption	0.333	0.125	7.033	0.008
ApoB	Body mass index	0.067	0.030	5.087	0.024
ApoAI/ApoB	Age	-0.021	0.007	9.644	0.002
	Alcohol consumption	-0.355	0.123	8.280	0.004
	Body mass index	-0.124	0.033	14.076	0.000

TC, total cholesterol; TG, triglyceride; HDL-C, high-density lipoprotein cholesterol; LDL-C, low-density lipoprotein cholesterol; ApoAI, apolipoprotein AI; ApoB, apolipoprotein B; ApoAI/ApoB, the ratio of apolipoprotein AI to apolipoprotein B

## Discussion

The present study shows that the levels of serum TC, HDL-C, LDL-C, ApoAI and ApoB were lower in Bai Ku Yao than in Han. There was no significant difference in the serum levels of TG and the ratio of ApoAI to ApoB between the two ethnic groups. These findings are consistent with those of our previous studies in a large population [7]. It is well known that dyslipidemia is a multifactorial origin, including hereditary and acquired risk factors. Bai Ku Yao is an isolated subgroup of the Yao minority in China. Strict intra-ethnic marriages have been performed in this population from time immemorial. Therefore, we hypothesized that the hereditary characteristics and genotypes of some lipid metabolism-related genes in this population may be different from those in Han Chinese.

The prevalence of the T677T genotype varies among different ethnic groups. It is very low in African populations, whereas in Europe and North America it ranges between 5% and 15%. In Italy an even higher prevalence has been reported in some regions [44]. The frequency of the MTHFR 677T allele in healthy controls was 13.4% among Saudi Arabian [45], 21.67% among Egjiptian [36], 28.75% among Tunisian Arabs [26], 35.02% among the Greek (Attica region, the ATTICA study) [20], 47.9% (Ashkenazi Jews) and 18.1% (Yemenite Jews) among Israeli [46]. MTHFR 677T variant was detected in only 20% of Black South Africans (no homozygotes) versus 56% of Caucasians with 12% homozygotes (*P *< 0.0001) [31]. It has been reported that the homozygocity for the T allele of MTHFR was more frequent in patients with premature myocardial infarction than in controls (27.1% vs. 14.6%, *P *= 0.02) [30], in CAD patients than in control subjects (28.5% vs. 13.5%, *P *< 0.00003) [27], and in diabetics than in healthy subjects (12.8% vs. 7.2%) [23]. However, there was no significant difference in genotypic or allelic frequencies of the MTHFR C677T between hemodialysis patients and healthy controls [36], between hyperlipidemic and control groups [24], and between carotid stenosis patients and control subjects [22]. In the present study, we showed that the frequency of MTHFR 677T allele was lower in Bai Ku Yao than in Han. The frequency of CT and TT genotypes was also lower in Bai Ku Yao than in Han. These results indicate that the allelic variation of the MTHFR C677T may have an ethnic specificity.

The association of MTHFR C677T polymorphism and plasma or serum lipid profiles in different populations has been evaluated in several studies. However, the results are inconsistent. The MTHFR 677T allele has been found to be associated with unfavorable serum lipid profiles [18-26] and increased risk of CAD in some studies [26-30] but not in others [31-36]. It has been reported that the subjects with the T allele had the highest levels of TC and LDL-C, and the subjects with the CC genotype had the lowest [19]. Pitsavos *et al*. found that the oxidized (ox)-LDL levels were higher in TT genotype as compared to CC and CT genotypes. Mediterranean diet was associated with lower ox-LDL levels in TT and CT individuals, but not in CC subjects, after controlling for several potential confounders [20]. CAD patients with the MTHFR TT genotype had higher cholesterol and TG concentrations than patients with the MTHFR CC genotype [26]. TC and TG levels were statistically different in all MTHFR genotypes and in TC/TT groups [22]. The hyperlipidemia patients with MTHFR CT/TT genotype had a higher concentration of TG than those with CC genotype [24]. Real *et al*. [25] found significant differences in plasma HDL-C (CC: 1.39 ± 0.34, CT: 1.33 ± 0.39 and TT: 1.14 ± 0.26 mmol/L, *P *= 0.028) between the C677T MTHFR genotypes, that were also found when sex, age, and BMI were included as covariables. A significant correlation was also found between plasma homocysteine values and plasma HDL-C, but no correlations were found with age, BMI or other lipid and ApoB plasma values. However, Spiridonova *et al*. [33] showed that the MTHFR gene polymorphism was not associated with variation in either TC, very-low-density-lipoprotein cholesterol (VLDL), LDL-C, HDL-C, or TG levels in CAD patients and control subjects. Homocysteine did not correlate with TC, LDL, HDL, VLDL and TG in obese children and adolescents [32]. In the present study, we found that the levels of TC and LDL-C were significant differences among the three genotypes in both Bai Ku Yao and Han populations, the T allele carriers had higher serum TC and LDL-C levels as compared with the T allele noncarriers. The levels of ApoB in Han were also significant differences among the three genotypes, the T allele carriers had higher serum ApoB levels than the T allele noncarriers. Multivariate logistic regression analysis showed that the levels of TC, TG and LDL-C in Bai Ku Yao and LDL-C in Han were correlated with genotypes. These data suggest that the MTHFR C677T polymorphism was mainly associated with serum TC and LDL-C levels in our study populations, and might also involve in serum TG levels in Bai Ku Yao, and serum ApoB levels in Han. The reason for this discrepancy between the two ethnic groups may relate to the difference in the MTHFR gene-enviromental interactions [12].

In the present study, we also found that several environmental factors affect serum lipid levels. Serum lipid parameters were correlated with age, sex, alcohol consumption, cigarette smoking, BMI, and blood pressure. These findings suggest that the environmental factors also play an important role in determining serum lipid levels in these populations [7,8]. There was significant difference in diet and lifestyle between the two ethnic groups. Corn was the staple food and rice, soy, buckwheat, sweet potato, and pumpkin products were the subsidiary foods in Bai Ku Yao. Approximately 90% of the beverages were corn wine and rum. The alcohol content is about 15% (v/v). They are also accustomed to drink Hempseed soup. In contrast, rice was the staple food and corn, broomcorn, potato, and taro products were the subsidiary foods in Han. About 90% of the beverage was rice wine. The content of alcohol is about 30% (v/v). The staple and subsidiary foods are more favorable for serum lipid profiles in Bai Ku Yao than in Han. Corn contains abundant dietary fiber and plant protein [47]. Consumption of dietary fiber, specifically the soluble type, such as pectins and guar gum can decrease serum TC levels [48,49]. Plant protein might promote the transportation and excretion of free cholesterol. Dietary soy protein has well-documented beneficial effects on serum lipid concentrations [50,51]. Buckwheat protein product has a potent hypocholesterolemic activity [52,53]. Ludvik *et al*. [54] found that ingestion of 4 g/day caiapo (the extract of white-skinned sweet potato) for 6 weeks reduces TC and LDL-C in type 2 diabetic patients previously treated by diet alone. Studies have demonstrated that pumpkin is a useful therapy for hypercholesterolemia through reducing oxidative stress and cholesterol levels [55]. There are more than 29 fat soluble constituents in Hempseed, among which saturated and unsaturated fatty acid methyl esters account for 12.36% and 86.96%; respectively [56,57]. Several experimental and clinical studies have demonstrated that Hempseed or Hempseed oil can decrease TC, TG and LDL-C levels, inhibit lipid peroxidation, and reduce atherogenic index [58-62].

## Conclusion

The present study shows that there was significant difference in the genotypic and allelic frequencies of MTHFR C677T polymorphism between the Bai Ku Yao and Han populations. The frequency of MTHFR 677T allele and CT and TT genotypes was lower in Bai Ku Yao than in Han. The T allele carriers in both ethnic groups had higher serum TC and LDL-C levels as compared with the T allele noncarriers. The T allele carriers in Han had higher serum ApoB levels than the T allele noncarriers. Multivariate logistic regression analysis showed that the levels of TC, TG and LDL-C in Bai Ku Yao and LDL-C in Han were correlated with genotypes. The differences in serum TC, TG, LDL-C and ApoB levels between the two ethnic groups might partly result from different genotypic and allelic frequencies of the MTHFR C677T or different MTHFR gene-enviromental interactions.

## Competing interests

The authors declare that they have no competing interests.

## Authors' contributions

LZ participated in the design, undertook genotyping, and helped to draft the manuscript. RXY conceived the study, participated in the design, carried out the epidemiological survey, collected the samples, and drafted the manuscript. WYL, LM, DFW, LHHA, XJH and XLC collaborated to the genotyping. JZW and SLP carried out the epidemiological survey, collected the samples, and helped to carry out the genotyping. All authors read and approved the final manuscript.
